# Cholesterol Quantification in Brain and Spinal Cord During Development, Demyelination, and Remyelination

**DOI:** 10.3390/molecules31173033

**Published:** 2026-08-28

**Authors:** Disni Dedunupitiya, Eden P. Go, Travis Witte, Ashlynn Elliott, Nishama De Silva Mohotti, Jenna M. Williams, Hiroko Kobayashi, Rashmi Binjawadagi, Heather Desaire, Meredith D. Hartley

**Affiliations:** Department of Chemistry, University of Kansas, 1567 Irving Hill Road, Lawrence, KS 66045, USA; disni.dedunupitiya@ku.edu (D.D.); edenp@ku.edu (E.P.G.); tmwitte@ku.edu (T.W.); nishama@ku.edu (N.D.S.M.); hdesaire@ku.edu (H.D.)

**Keywords:** multiple sclerosis, myelin, demyelination, remyelination, myelin repair, cholesterol, cholesterol esters, gas chromatography mass spectrometry (GC-MS), liquid chromatography–mass spectrometry (LC-MS)

## Abstract

Cholesterol in the central nervous system (CNS) is largely unesterified (>99%) and is predominantly present in the myelin sheath (~70% of total CNS cholesterol). However, cholesterol ester accumulation has been observed during neurological disease. In this cross-sectional study, we measured free cholesterol and cholesterol ester levels in the brains and the spinal cords of *Plp1*-iCKO-*Myrf* mouse model during postnatal myelination, demyelination, and remyelination using gas chromatography–mass spectrometry with single ion monitoring (GC-MS-SIM) and liquid chromatography–mass spectrometry (LC-MS). Cholesterol levels in healthy mouse brains increased steadily up to 38 weeks of age. In contrast, cholesterol in the healthy spinal cord increased until P42, and then remained steady out to 38 weeks of age. During demyelination, both brain and spinal cord cholesterol levels were significantly reduced compared to healthy mice and did not return to normal cholesterol levels even during remyelination. Semi-quantitative quantification of cholesterol esters during peak demyelination revealed that cholesterol esters comprise 19% of the measured cholesterol pool in the brain and 66% in the spinal cord. Robust methods for internal standard-based quantification of CNS cholesterol and cholesterol esters were developed, which are critical for revealing mechanisms of cholesterol regulation during disease.

## 1. Introduction

Cholesterol plays critical structural, biophysical, and metabolic roles in the central nervous system (CNS). CNS tissue contains approximately 20% of the whole-body free cholesterol pool, although it comprises only 2% of the body mass [1,2]. De novo synthesis of cholesterol is the primary source of cholesterol in the CNS, and the blood–brain barrier blocks entry of peripheral cholesterol into the brain under normal physiological conditions. Brain cholesterol is a structural component of myelin (~70% of total brain cholesterol) and of glial and neuronal cell membranes (~30%) [3]. The myelin sheath is a lipid-rich multilamellar structure that wraps segments of neurons, improving the efficiency of the rapid conduction of nerve impulses. Myelin contains a higher percentage of lipids (~80% lipids, 20% proteins) relative to typical cellular membranes (~40% lipids, 60% proteins) [4,5]. The higher proportion of lipids in myelin causes a more tightly packed arrangement of the membrane than in regular biological membranes [6].

Myelin is formed by the extension of oligodendrocyte membranes in the CNS, and cholesterol biosynthesis in oligodendrocytes is a rate-limiting factor in myelin membrane growth during brain development and maturation. Mice that lack cholesterol synthesis in myelinating oligodendrocytes showed severely perturbed myelination and suffered from ataxia and tremor [7]. Most cholesterol in the brain is unesterified, with <1% of total cholesterol existing in the esterified form as cholesterol esters [8]. Cholesterol comprises ~40% of myelin lipids and is a key structural component of the myelin membrane that contributes to myelin membrane stability [9]. Repaired myelin showed reduced biophysical stability relative to healthy myelin, which was due in part to reduced cholesterol levels in repaired myelin [10]. Cholesterol also plays a critical role as a metabolic precursor for neuroactive steroids, which regulate myelin formation, regeneration, and neuroprotection [11]. 

Disruption of CNS cholesterol homeostasis has been observed in many neurological diseases, suggesting an interconnection between neuronal health and cholesterol [12]. Due to the high levels of cholesterol in myelin, diseases featuring loss of myelin have been linked to cholesterol metabolism and regulation. The most prevalent neurodegenerative disease with primary myelin damage is multiple sclerosis (MS), in which inflammatory demyelination leads to degeneration of neurons in the CNS [13]. Blocking cholesterol synthesis in either oligodendrocytes [7] or neurons [14] greatly impaired remyelination in mouse models of demyelination, underscoring the requirement of cholesterol for myelin repair. The accumulation of cholesterol esters is associated with myelin damage and has been observed in brain white matter, spinal cords, and cerebrospinal fluid of people who were diagnosed with multiple sclerosis [15,16,17]. Elevated cholesterol esters during demyelination have also been observed in preclinical demyelination models including the cuprizone model [18] and experimental autoimmune encephalomyelitis (EAE) model [19]. In our previous study, we observed significant accumulation of cholesterol esters in a genetic mouse model of demyelination (*Plp1*-iCKO-*Myrf*) [20]. 

Acetyl-CoA-acyltransferase 1 (ACAT1) and lecithin–cholesterol acyltransferase (LCAT) are CNS enzymes that can convert cholesterol to cholesterol esters. Both ACAT1 and LCAT were elevated in *Plp1*-iCKO-*Myrf* brain and spinal cord tissues [20]. Microglia are critical glia in the CNS that respond to demyelination by phagocytosing myelin debris, and ACAT1 is expressed by microglia [21]. Lending support to a role for ACAT1 in cholesterol ester formation in the *Plp1*-iCKO-*Myrf* model, lipid droplet accumulation was observed in microglia during peak demyelination. LCAT is an extracellular enzyme that is secreted by astrocytes and acts on cholesterol in lipoprotein particles [22]. LCAT is not well studied in the context of demyelination, but it showed greater upregulation than ACAT1 in the *Plp1*-iCKO-*Myrf* model. 

Another relevant feature of the *Plp1*-iCKO-*Myrf* model is that the brain and spinal cord showed differences in terms of both cholesterol esters and remyelination [20,23]. During demyelination, the brain showed increased cholesterol esters that returned to normal levels during robust remyelination. In contrast, the spinal cord had chronic accumulation of cholesterol esters, limited remyelination, and persistent microglial activation [24]. The previous study, however, lacked a quantitative assessment of cholesterol levels in the brains and spinal cords of *Plp1*-iCKO-*Myrf* mice during the full disease course.

In this study, we profiled the brain and spinal cord levels of free cholesterol and cholesterol esters during development, demyelination, and remyelination in the *Plp1*-iCKO-*Myrf* mouse model using robust, internal standard-based quantification methods. Most prior studies have focused on brain cholesterol, but here we simultaneously examined both brain and spinal cord levels, as the tissues differ in myelin development, anatomy, and responses to myelin damage. During development, we observed steady increases in free cholesterol levels in both the brain and the spinal cord. Cholesterol ester levels were highest at P1 in the spinal cord and decreased rapidly by P42, whereas the developmental brain had similar levels of cholesterol esters throughout. Following healthy mice out to 38 weeks, cholesterol levels continued to increase in the brain but plateaued in the spinal cord. In adult mice, the spinal cord had ~2-fold more cholesterol per tissue weight compared to the brain. When demyelination was induced in adult mice, cholesterol levels in both the brain and the spinal cord dropped and did not return to healthy levels even after the damaged myelin was repaired. A quantitative assessment of cholesterol esters revealed that 19% of the measured cholesterol pool was esterified in the brain during peak demyelination and 66% was esterified in the spinal cord. Our results suggest that demyelination in this model chronically reduces the CNS levels of total free cholesterol and establishes a robust, quantitative set of methods for measuring free cholesterol and cholesterol esters in the CNS. Reliable analytical methods are critical for revealing mechanistic insights into how cholesterol metabolism is related to the pathophysiology of neurological diseases and may enable the discovery of new therapeutic targets for disease.

## 2. Results

### 2.1. MS Methods for Detecting Cholesterol and Cholesterol Ester Levels in CNS Tissue Homogenate Samples

**Figure 1:**
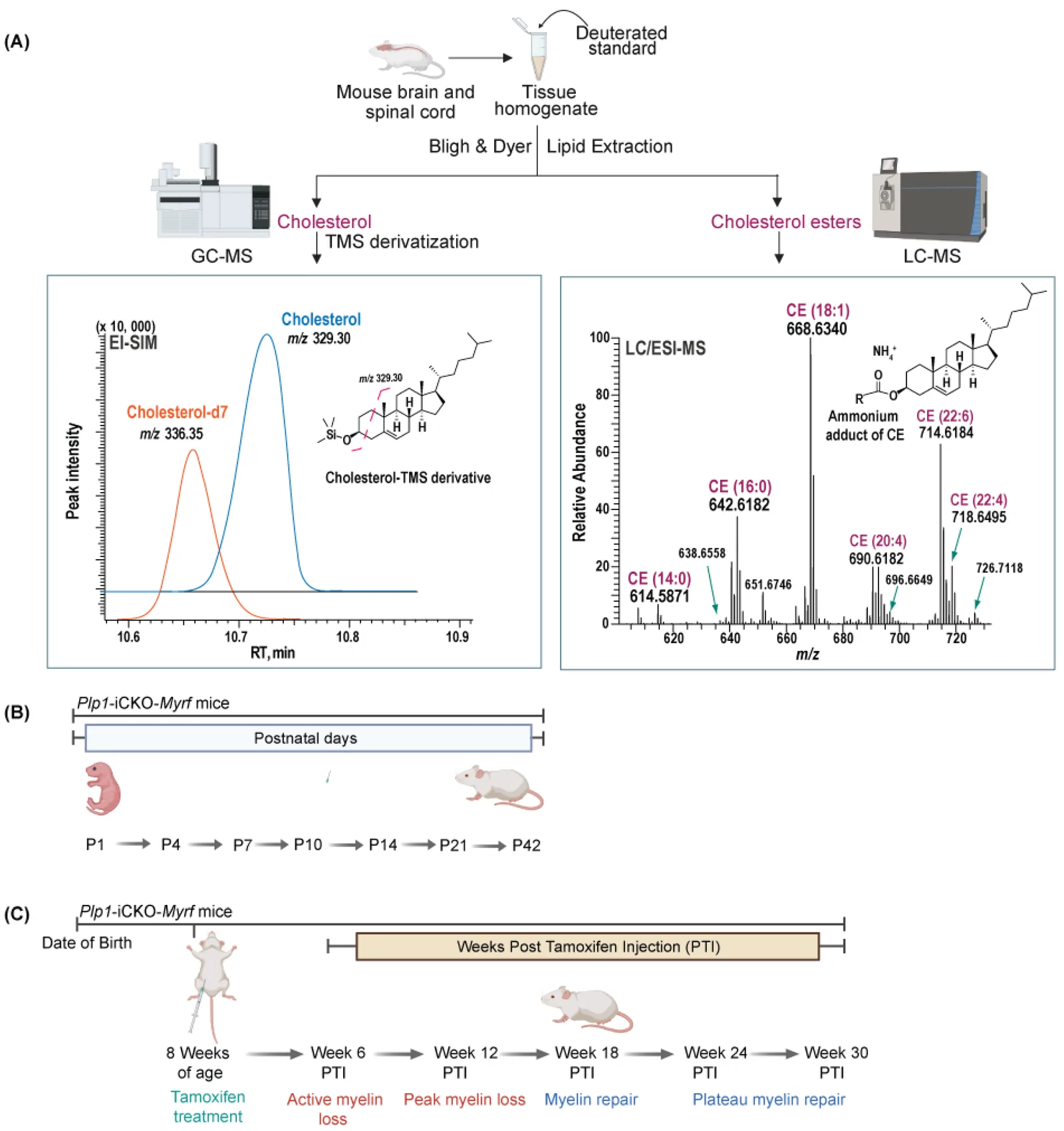
Overview of the experimental workflow for cholesterol analysis. (**A**) Experimental workflow for the quantification of cholesterol and cholesterol esters (CE) using GC-MS and LC-MS. (**B**) Tissue collection time points of mice during development (P1–P42). (**C**) Timeline of the *Plp1*-iCKO-*Myrf* mouse model of demyelination and tissue collection time points.

To accurately measure cholesterol in CNS tissue samples, we developed a quantitative method with an internal standard using GC-MS with electron ionization and single ion monitoring [25,26]. In brief, the method involves collection of brains and spinal cords, tissue homogenization, lipid extraction, lipid extract derivatization, and GC-MS sample analysis (Figure 1A). Each sample was spiked with deuterated cholesterol (d_7_-cholesterol) at the beginning of the lipid extraction, which enables more accurate quantification of the cholesterol levels in the tissues.

To derivatize the cholesterol prior to GC-MS analysis, N,O-Bis(trimethylsilyl)trifluoroacetamide with trimethylchlorosilane (BSTFA + TMCS, 99:1, *v*/*v*) was used to modify the hydroxyl of cholesterol with trimethylsilyl (TMS) to increase the volatility of the molecule. TMS-derivatization was chosen over other derivatization methods because the reaction was straightforward and generated more volatile and less polar derivatives with structurally informative fragments [27]. The TMS-derivative of cholesterol ionized by electron ionization and yielded highly abundant, reproducible fragments (*m*/*z* 329, *m*/*z* 336) of cholesterol and d_7_-cholesterol, respectively, which were used for quantification (Figure 1A). 

Cholesterol ester levels were quantified using ultra-high-pressure liquid chromatography–electrospray ionization–mass spectrometry (UPLC-ESI-MS) [28]. Unlike cholesterol, cholesterol esters form stable ammonium adduct ions in the ESI source and can be detected by UPLC-ESI-MS (Figure 1A).

### 2.2. Trends of Free Cholesterol and Cholesterol Ester Levels in the Developmental Brain and Spinal Cord

**Figure 2:**
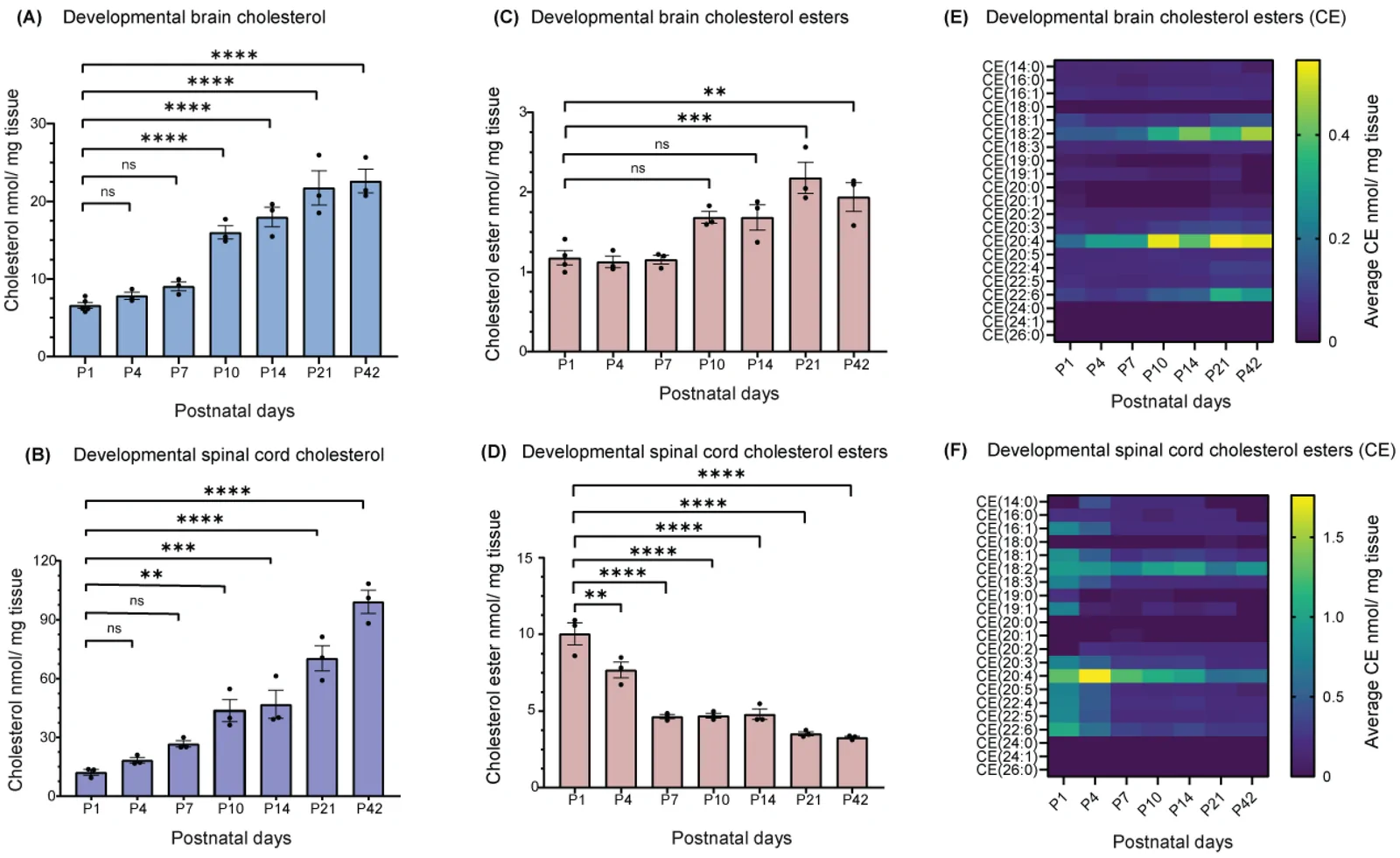
Overview of cholesterol increases with age during development in the brain and spinal cord. (**A**) Total free cholesterol levels in the P1–P42 mouse brain. (**B**) Total free cholesterol levels in the mouse P1–P42 spinal cord. Ordinary one-way ANOVA, *n* = 3–5, Dunnett’s test for multiple comparisons are plotted as mean ± SEM, ns ≥ 0.05; ** *p* < 0.01; *** *p* < 0.001; **** *p* < 0.0001. (**C**–**F**) Cholesterol esters are accumulated during the early developmental spinal cord whereas they are accumulated at the later developmental time points in the brain. (**C**) Total cholesterol esters in the P1–P42 mouse brain. (**D**) Total cholesterol esters in the mouse P1–P42 spinal cord. Ordinary one-way ANOVA, *n* = 3–5, Dunnett’s test for multiple comparisons. Data are plotted as mean ± SEM, ns ≥ 0.05; ** *p* < 0.01; *** *p* < 0.001; **** *p* < 0.0001. (**E**) Heat map showing average individual cholesterol ester concentrations (nmol/mg tissue) in the developmental brain (P1–P42). (**F**) Heat map showing average individual cholesterol ester concentration (nmol/mg tissue) in the developmental spinal cord (P1–P42).

To determine the physiological levels of free cholesterol during CNS development, we collected brain and spinal cord tissues at postnatal days P1, P4, P7, P10, P14, P21, and P42 (Figure 1B). In the brain, the cholesterol concentration was 7 nmol/mg tissue at P1 and increased to 23 nmol/mg at P42, a ~3.4-fold increase (Figure 2A, Supplementary Table S1). The spinal cord tissue showed a more dramatic increase in cholesterol starting at 12 nmol/mg at P1 and increasing to 99 nmol/mg by P42, a ~8.1-fold increase (Figure 2B, Supplementary Table S2).

We detected 21 cholesterol esters, with fatty acyl chains ranging from 14:0 to 22:6 in the postnatal mouse brain and spinal cord tissues (Supplementary Tables S7 and S9). Total cholesterol ester levels in the brain ranged from 1 nmol/mg to 2 nmol/mg (Figure 2C,E, Supplementary Table S7). The spinal cord tissue had an average of 10 nmol/mg cholesterol esters at P1 and decreased to 3 nmol/mg by P42 (Figure 2D,F, Supplementary Table S9). The spinal cord had higher levels of total cholesterol esters as compared to the brain at developmental time points (8.6-fold higher than in the brain at P1, dropping to 1.8-fold by P42). In summary, total free cholesterol levels increased with developmental age in both the brain and the spinal cord; however, only the spinal cord had a high accumulation of cholesterol esters at P1 that rapidly decreased by P42.

### 2.3. Cholesterol Increases with Adult Development in the Brain, but Not the Spinal Cord

In this study, we utilized a genetic mouse model of demyelination (*Plp1-CreERT*; *Myrf(fl/fl)* or *Plp1*-iCKO-*Myrf*) to measure the dynamic changes in cholesterol over time in the brain and spinal cord (Figure 1C). Demyelination was induced with tamoxifen injections at 8 weeks of age, which caused ablation of *Myrf* (myelin regulatory factor) from mature oligodendrocytes. MYRF is a transcription factor that regulates the transcription of genes important in the production and maintenance of myelin [29]. Deletion of *Myrf* results in oligodendrocyte cell death and gradual demyelination; however, *Myrf* is unaffected in oligodendrocyte precursor cells (OPCs). Following demyelination, OPCs proliferate and differentiate into mature myelinating oligodendrocytes, leading to remyelination [30,31]. Prior studies have demonstrated that loss of myelin led to motor impairment measured by clinical scoring and rotarod analysis that was detectable by 5 weeks post-tamoxifen and peaked at 10–14 weeks post-tamoxifen. Motor symptoms gradually improved from 14 to 24 weeks post-tamoxifen [23,31].

**Figure 3:**
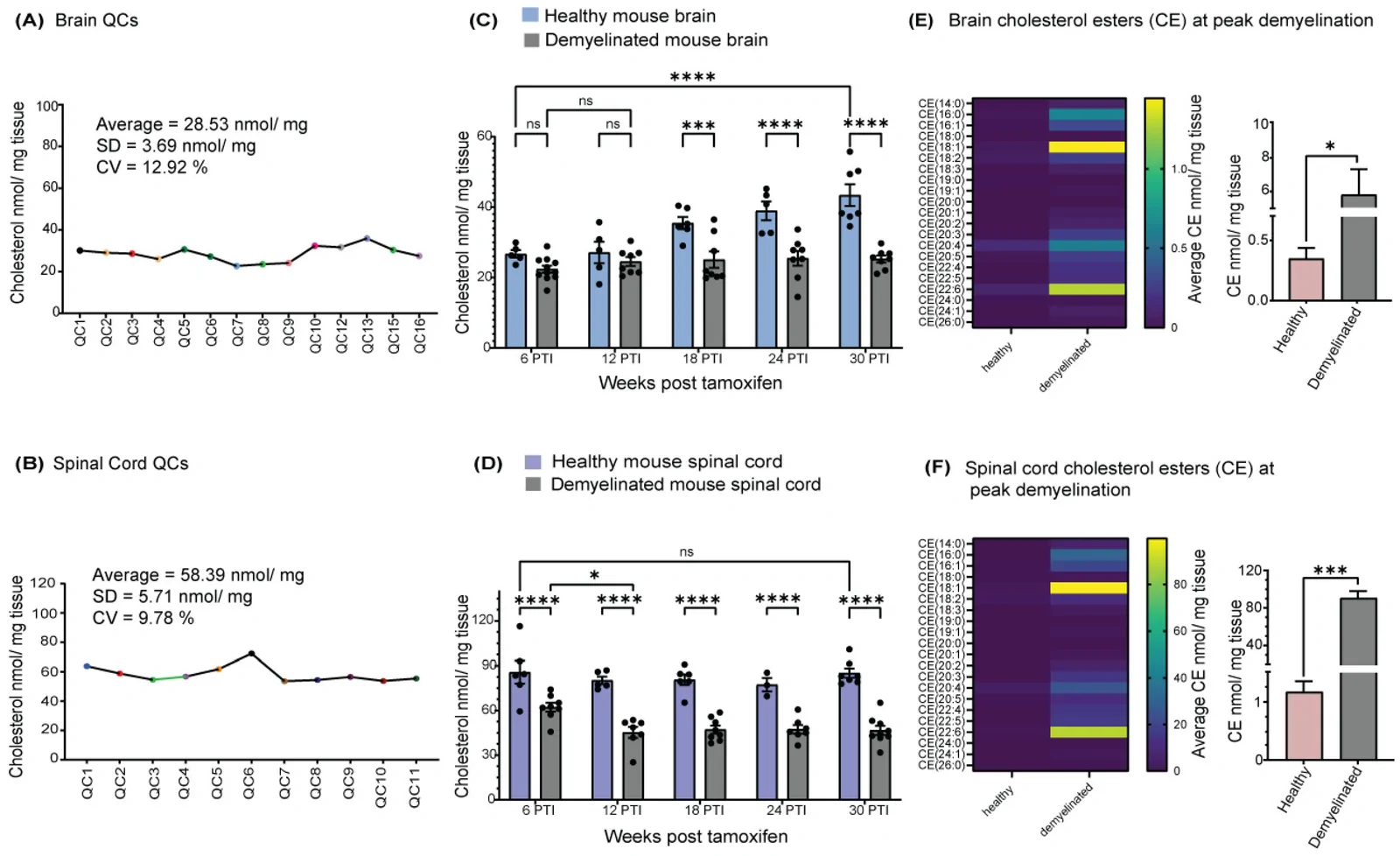
Total free cholesterol levels are reduced in both the brain and spinal cord in demyelinated mice. (**A**) Brain quality control samples (QCs) showed an average of 28.53 nmol cholesterol per mg of tissue with a coefficient of variation (CV) of 12.92%; SD—standard deviation. (**B**) Spinal cord quality control samples (QCs) showed an average of 58.39 nmol cholesterol per mg tissue with a coefficient of variation (CV) of 9.78%. (**C**) Total free cholesterol levels in the *Plp1*-iCKO-*Myrf* mouse brain. Two- way ANOVA, Tukey’s correction for multiple comparisons, *n* = 5–11, data are plotted as mean ± SEM, ns ≥ 0.05; *** *p* < 0.001; **** *p* < 0.0001. (**D**) Total free cholesterol levels in the *Plp1*-iCKO-*Myrf* mouse spinal cord. Two-way ANOVA, Tukey’s correction for multiple comparisons, *n* = 3–9, data are plotted as mean ± SEM, ns ≥ 0.05, * *p* < 0.05; **** *p* < 0.0001. (**E**,**F**) Cholesterol esters were increased at the peak demyelination time points of the brain and spinal cord. (**E**) Total amounts of cholesterol ester (CE) and individual CE types (heat map, average CE nmol/mg tissue) in the adult healthy and demyelinated mouse brain at week 12 post-tamoxifen. (**F**) Total amounts of cholesterol ester (CE) and individual CE types (heat map, average CE nmol/mg tissue) in the adult healthy and demyelinated mouse spinal cord at week 18 post-tamoxifen. Unpaired, two-tailed *t*-test was performed for the bar graphs in (**E**,**F**); CE data are plotted as mean ± SEM, * *p* < 0.05; *** *p* < 0.001.

For the demyelination experiment, we collected tissues from 70 to 80 mice. TMS-derivatized cholesterol is only stable for ~48 h, which limited the number of samples that could be derivatized and analyzed in a single batch for GC-MS cholesterol analysis. To overcome this problem, the samples were randomly divided into multiple batches to perform the lipid extraction, sample derivatization, and GC-MS analysis. Since GC-MS runs were performed on different days, separate internal standard calibration curves were prepared to avoid instrumental variations. The LOD and LOQ for cholesterol (1.10 μM and 3.69 μM, respectively) were determined using the method of standard deviation. To determine variation across batches, two or three quality control samples were analyzed with each batch. Quality control samples were prepared by mixing representative brain or spinal cord samples from each group. In Figure 3A, inter-assay brain quality control samples showed an average of 29 nmol/mg tissue concentration with a 12.9% coefficient of variation (CV). Spinal cord quality control samples were 59 nmol/mg with 9.8% CV (Figure 3B). These CV values exhibited less than 15%, meeting the acceptance criteria recommended for validated bioanalytical assays [32]. Intra-assay accuracy and precision are shown by the QC run within one batch, and those values are included in Supplementary Table S13.

In healthy mouse brains (*Cre*-negative *Plp1*-iCKO-*Myrf* mice), total free cholesterol was 27 nmol/mg tissue at 14 weeks of age (week 6 post-tamoxifen) and reached 43 nmol/mg tissue by 38 weeks of age (week 30 post-tamoxifen) (Figure 3C, Supplementary Table S3). In the spinal cord, cholesterol levels remained relatively stable in the range of 77 nmol/mg to 86 nmol/mg tissue throughout all time points (14–38 weeks of age) (Figure 3D, Supplementary Table S5). Thus, cholesterol levels were still increasing in the brains of mice as old as 38 weeks, which is approaching “middle-aged” for a mouse [33]. In contrast, cholesterol levels in the spinal cord were at the same level from 14 to 38 weeks of age, suggesting that cholesterol metabolism reached a steady state homeostasis by ~3 months of age. At week 6 post-tamoxifen, brain cholesterol was 3-fold lower than in the spinal cord; however, as the cholesterol levels in the brain continued to increase, the levels were only 2-fold lower than the spinal cord at 30 weeks post-tamoxifen (38 weeks of age) (Figure 3C,D, Supplementary Tables S3 and S5). Even with the increase in brain cholesterol, the spinal cord still had higher cholesterol consistent with higher myelin density [2,23].

### 2.4. Brain and Spinal Cord Free Cholesterol Levels Are Impaired with Demyelination

The cholesterol levels in the brains of *Plp1*-iCKO-*Myrf* mice were measured during active demyelination (week 6 post-tamoxifen), peak demyelination (week 12 post-tamoxifen), initial recovery (week 18 post-tamoxifen), and plateau recovery (weeks 24 and 30 post-tamoxifen) (Figure 3C). Strikingly, the total free cholesterol did not drop significantly with the onset of demyelination at week 6 and remained consistent at ~25 nmol/mg tissue during all subsequent time points, even during the remyelination period. Since the total free cholesterol levels in the healthy brains increased over that same time period, the brains at weeks 18, 24, and 30 post-tamoxifen had significant depletion in total cholesterol (Figure 3C, Supplementary Table S3) [31]. These results suggest that widespread demyelination leads to a persistent reduction in brain cholesterol relative to healthy adult mice even during robust remyelination at the later time points. 

In the spinal cord, cholesterol levels were significantly reduced in the demyelinated mice relative to healthy mice at all time points measured. Healthy spinal cord tissue at week 6 post-tamoxifen had an average of 85 nmol/mg tissue, and with demyelination, the cholesterol levels at week 6 dropped to 62 nmol/mg tissue and continued to decrease to the range of ~45 nmol/mg tissue that persisted through the remaining time points (Figure 3D, Supplementary Table S5). By the end of the experiment (week 30 post-tamoxifen), both the brain at 42% and the spinal cord at 45% showed similar levels of cholesterol depletion relative to healthy tissues (Supplementary Tables S3 and S5).

In our previous study, we measured the levels of cholesterol esters in the *Plp1*-iCKO-*Myrf* mice across the same time points examined here, and we observed major increases in cholesterol esters during peak demyelination over healthy tissues (30-fold in the brain and 130-fold in the spinal cord) [20]. In that study, cholesterol esters were measured by direct infusion of the extracted lipids into a mass spectrometer with no chromatographic separation. A semi-quantitative method was used based on internal standards spiked at known amounts, but a calibration curve was not prepared, and thus the amounts reported were relative values. When we compared the cholesterol ester data obtained from our current quantitative method to the previous direct infusion method, we noticed that the levels of cholesterol esters detected in the healthy tissue in the developmental study were several fold higher than the healthy levels we previously reported, suggesting that our previous measurements lacked quantitative accuracy. Thus, to obtain a more accurate quantification of cholesterol esters during peak demyelination in the *Plp1*-iCKO-*Myrf* mice, we applied our new quantitative method including a calibration curve to samples from peak demyelination in the brain (week 12) and in the spinal cord (week 18). In healthy brains, the cholesterol ester levels were ~0.3 nmol/mg tissue, which increased to ~6 nmol/mg (16-fold increase) during demyelination. The healthy spinal cord had ~1 nmol/mg cholesterol esters, which was more dramatically elevated 77-fold to 91 nmol/mg during demyelination (Figure 3E,F, Supplementary Tables S11 and S12).

### 2.5. Thyroid Hormone Action Does Not Deplete Total Free Cholesterol Levels in the CNS

Sob-AM2 is a CNS-penetrating prodrug that can cross the blood–brain barrier to enter the central nervous system, where it is hydrolyzed to the parent thyroid hormone agonist known as sobetirome [34,35]. Sob-AM2 has previously been shown to improve remyelination and motor recovery in the *Plp1*-iCKO-*Myrf* demyelination model [31]. In peripheral tissues, thyroid hormone is a well-established regulator of cholesterol metabolism, and increased thyroid hormone lowers serum cholesterol through liver clearance mechanisms [36]. Reduced cholesterol in the CNS is associated with impaired remyelination [7,37], and therefore, we tested how Sob-AM2 affected total cholesterol levels in the brain and spinal cord. 

**Figure 4:**
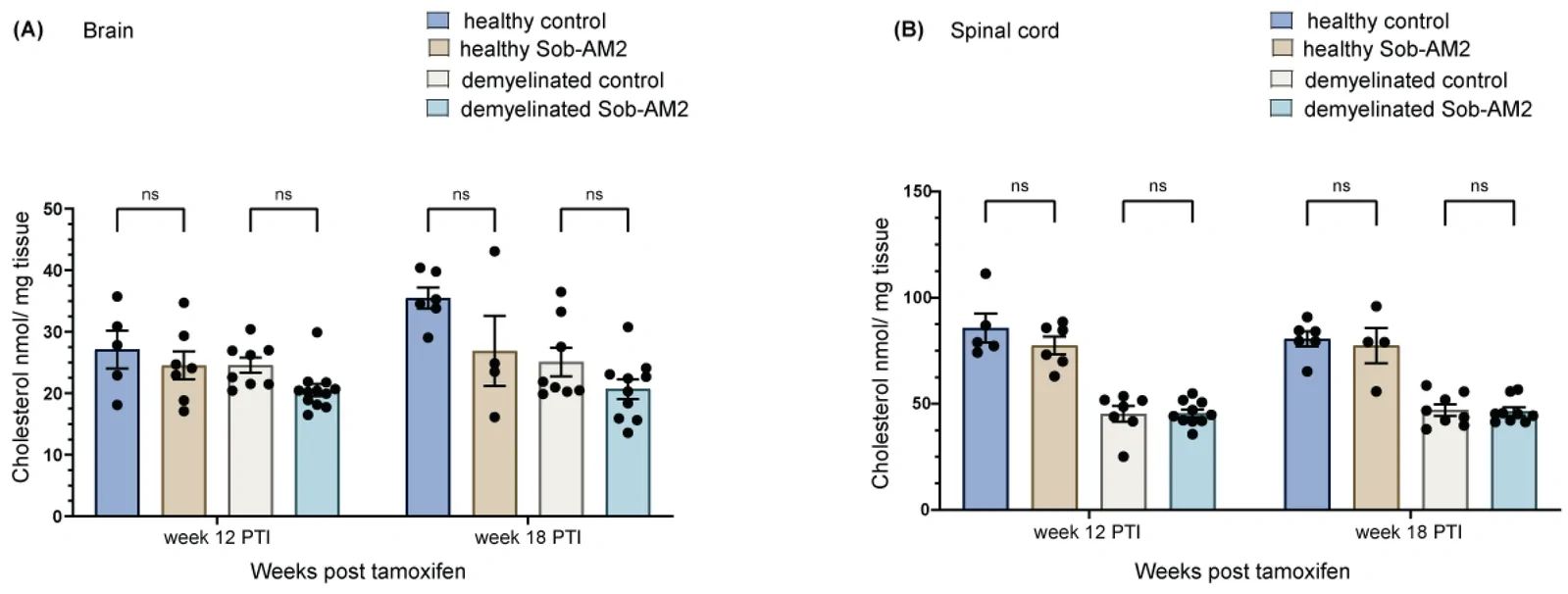
Sob-AM2 treatment does not significantly alter total CNS free cholesterol levels in *Plp1*-iCKO-*Myrf* mice. (**A**) Effect of the Sob-AM2 drug on cholesterol levels in the brain. (**B**) Effect of the Sob-AM2 drug on cholesterol levels in the spinal cord. Three-way ANOVA with the Šídák correction for multiple comparisons *n* = 4–10; data are plotted as mean ± SEM; ns ≥ 0.05.

Mice were treated with chow containing Sob-AM2 starting 2 weeks post-tamoxifen. Tissue samples were analyzed from week 12 post-tamoxifen (peak demyelination) and week 18 post-tamoxifen (recovery). Overall, the mice treated with SobAM2 showed similar trends to the vehicle mice with reduced cholesterol levels in demyelinated mice as compared to healthy mice in both brains (Figure 4A, Supplementary Table S4) and spinal cords (Figure 4B, Supplementary Table S6). No differences were observed due to drug treatment. Considering the critical role of cholesterol in CNS myelination and remyelination, our observation that thyroid hormone agonists do not reduce brain cholesterol in healthy brains or chronically in the context of demyelination is additional support for the therapeutic potential of Sob-AM2, which has shown benefit in preclinical models of demyelinating diseases [31,38].

## 3. Discussion

The brain is considered the most cholesterol-rich organ (15–20 mg/g tissue) containing 20% of the whole-body cholesterol [3,39]. Most of brain cholesterol (70%) is in the myelin sheath, where it is an essential structural component, but cholesterol also serves as a precursor of steroid hormones [40] and a required component of synapses [11]. Brain cholesterol metabolism is separated from peripheral cholesterol metabolism by the blood–brain barrier [39]. Brain cholesterol is primarily generated by astrocytes and is known to be transported by apolipoprotein E (apoE) to neurons [41]. However, both oligodendrocytes and neurons are also capable of de novo synthesis of cholesterol [7,14]. Brain cholesterol levels increase during the prenatal period and adolescence, correlating with the increase in myelin levels. In the adult brain, cholesterol has low turnover with a long half-life [3]. Excess cholesterol in the brain is esterified (1%) and stored as lipid droplets inside cells [8], or converted to oxysterols that can cross the blood–brain barrier [42].

Although lipidomics studies on CNS tissues are becoming more common, it remains challenging to accurately quantify cholesterol and related sterols using mass spectrometry methods reliant on electrospray ionization (ESI) as cholesterol does not ionize well under ESI conditions. Cholesterol quantification via derivatization and analysis by GC-MS remains the most robust method for absolute quantification. Since cholesterol is such a critical component of the CNS, it is critical that robust analytical methods like those developed in this study are used to enable mechanistic insight into the role of cholesterol during CNS development and disease. 

Myelination begins in the rodent brain around P10, and the mass of the brain doubles and water content decreases during this phase. Following the initial rapid phase of myelination, slower myelination continues during the final brain maturation stages. Previous studies have suggested that cholesterol levels track with myelination. Cholesterol in the developing rat brain was 10.7 μmol/g wet weight (P3), 12.6 μmol/g (P6), and 22.6 μmol/g (P12) [43]. It has also been observed that rates of cholesterol synthesis in the CNS were high (app. 0.26 mg/day) in the CNS during first three weeks after birth, and the rate declined to a constant value (app. 0.035 mg/day) in older mice at 13–26 weeks old [44]. The total cholesterol levels in our data align with the brain developmental phases and the cholesterol levels previously described. We observed that cholesterol levels increased rapidly at P10, and then gradually increased until P42 in both the brain and spinal cord (Figure 2A,B, Supplementary Tables S1 and S2). In humans, prolonged myelination starts prenatally, whereas it mainly occurs postnatally in rodents [45]. Plasma cholesterol levels correlate between the human fetus and the mother at the fetus ages younger than 6 months, but after that point the fetus produces majority of the cholesterol by itself [46]. However, the specific time at which the blood–brain barrier becomes impermeable to cholesterol during brain development in humans has not been determined yet [45].

Following the developmental phase, we observed that total brain cholesterol levels in healthy mice increased from 14 to 38 weeks of age (Figure 3C, Supplementary Table S3), which is consistent with previous measurements of brain cholesterol [44,47]. Increasing cholesterol during adulthood also correlates with the ongoing myelination in the corpus callosum and cortex of mice that continues through adulthood [48,49,50].

In contrast to the brain, there are few previous studies that have quantified cholesterol levels in the spinal cord during development and adulthood. Our spinal cord data showed that cholesterol increased during development (up to P42) and then stabilized 6–30 weeks post-tamoxifen (14–38 weeks of age) (Figure 2B and Figure 3D, Supplementary Tables S2 and S5). Recent RNA sequencing analysis has indicated that the oligodendrocytes from the brain and spinal cord show differences in pathways associated with cholesterol biosynthesis [51]. During nervous system development and myelination, the spinal cord is fully and stably myelinated at an earlier stage than in the brain in both rodents and humans, and thus the spinal cord–cholesterol dynamics that we measured correlated with known myelination trends [52].

**Figure 5:**
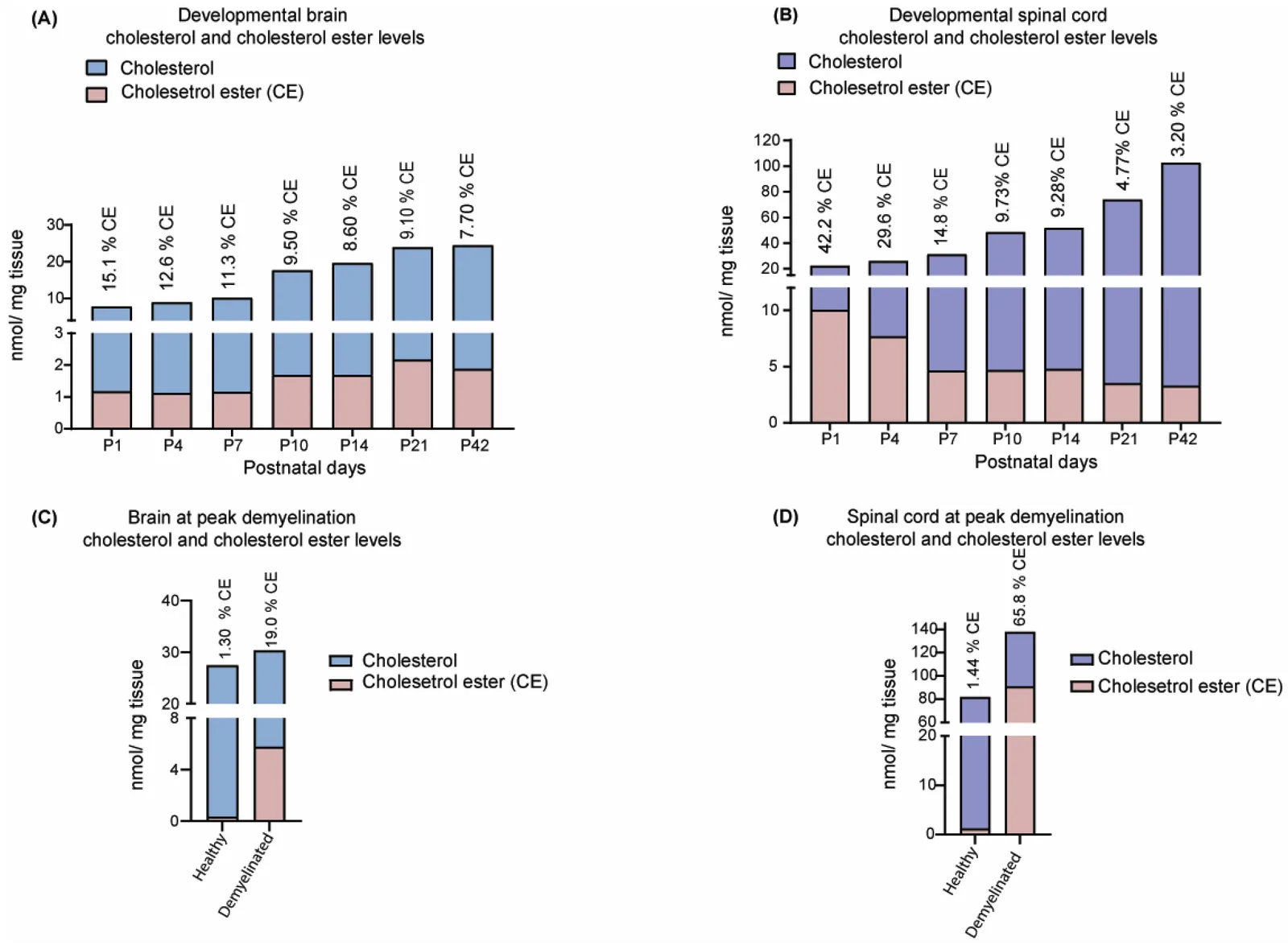
Percentage of cholesterol esters (CE) in the measured cholesterol pool decreases during the development and increases during demyelination in both the brain and the spinal cord. (**A**) Calculated CE percentage in the measured cholesterol pool in the developmental brain during P1–P42. (**B**) Calculated CE percentage in the measured cholesterol pool in the developmental spinal cord during P1–P42. (**C**) Calculated CE percentage in the measured cholesterol pool in the healthy and demyelinated brain at peak demyelination (week 12 post-tamoxifen). (**D**) Calculated CE percentage in the measured cholesterol pool in the healthy and demyelinated spinal cord at peak demyelination (week 18 post-tamoxifen). Average free cholesterol and cholesterol ester levels are plotted in the combined bar graphs above. Percentage CE was calculated as: CE/(CE + free cholesterol) × 100%. Please refer to Figure 2 and Figure 3 for all individual data points including error bars and statistics.

To analyze how the measured cholesterol pool changed, we compared the cholesterol and cholesterol ester levels in the brain and spinal cord during development. During development, the brain initially (P1–P7) had ~1 nmol/mg tissue of cholesterol esters (~15% of total pool), which increased to 1.6–2.0 nmol/mg levels by P10 (~7% of total pool). Since the free cholesterol levels continuously increased in the postnatal mouse brain, the percentage of cholesterol esters in the measured cholesterol pool decreased with age (Figure 5A, Supplementary Table S8). Notably, the spinal cord has a very high accumulation of cholesterol esters (10 nmol/mg at P1, ~45% of total pool) at early postnatal time points which decreased to 3.0 nmol/mg by P42 (~3% of total pool) (Figure 5B, Supplementary Table S10). The high level of cholesterol esters during early development could indicate that there is a large pool of excess cholesterol in spinal cord, which may be related to the high demand for cholesterol at this time point. In healthy brain and spinal cord during adulthood, cholesterol esters make up 1.0–1.5% of the measured cholesterol pool.

The pathology of multiple sclerosis is associated with altered cholesterol and cholesterol metabolite biomarkers in the serum or cerebrospinal fluid [13,53]; however, it still remains unclear whether peripheral dyslipidemia (high cholesterol and lipids) causes multiple sclerosis pathology or vice versa [54]. In human MS lesion samples, cholesterol synthesis gene expression was decreased in astrocytes and neurons, but increased in oligodendrocytes [14], suggesting cell-specific responses to inflammatory demyelination. In the brains of *Plp1*-iCKO-*Myrf* mice undergoing demyelination, we observed only a small loss of cholesterol at the initial time points (week 6 and week 12 post-tamoxifen). However, during the remyelination phase, total cholesterol levels remained steady (~25 nmol/mg) and did not increase with age as occurred in the healthy mice brains (Figure 3C, Supplementary Table S3). This result was somewhat surprising, as prior histological and motor testing in this model demonstrated increased myelin levels and improved motor performance at weeks 18 and 24 [23], which might be expected to correlate with increased cholesterol production. Previous studies have shown that the *Plp1*-iCKO-*Myrf* mouse model has robust remyelination in the brain with normal numbers of mature oligodendrocytes suggesting that the lack of cholesterol is not tied to defects in OPC differentiation [20,31]. The mechanistic basis for the reduced brain cholesterol is unclear; it may occur through disrupted cholesterol biosynthesis, increased cholesterol efflux [42], or increased cholesterol conversion to oxysterols [37] or other metabolites. Therefore, detailed isotope labeling studies and mass spectrometry analysis of cholesterol synthesis pathway intermediates, cholesterol metabolites, and oxysterols will be required to discern the mechanistic basis for the observed disruption in free cholesterol.

In contrast to the brain, the spinal cord had a much larger drop in total cholesterol during the initial demyelination phase (~26% loss). Following the initial drop, the spinal cord reached a steady plateau level during all time points (~45 nmol/mg tissue) (Supplementary Tables S3 and S5). Since the spinal cord cholesterol levels do not appreciably increase after 14 weeks of age, it is not surprising that total cholesterol did not increase after the primary demyelination insult. The loss of cholesterol with no subsequent increase is consistent with the limited remyelination observed in the spinal cord of this model [23].

Cholesterol is a critical component of myelin, which is underscored by studies showing that impaired cholesterol biosynthesis in both oligodendrocytes and neurons reduces remyelination [7,14]. Sob-AM2 is a CNS-penetrating thyroid hormone that has been previously shown to promote remyelination in multiple mouse models of demyelination including the *Plp1*-iCKO-*Myrf* mouse model used in this study [31,38]. In this study, we observed that Sob-AM2 treatment does not affect overall cholesterol levels (Figure 4). This suggests that Sob-AM2-mediated remyelination may not be mediated through direct changes to cholesterol biosynthesis. Myelin isolated from the *Plp1*-iCKO-*Myrf* mice brains at 24 weeks post-tamoxifen following demyelination had an approximate two-fold reduction in cholesterol levels [10], suggesting that repaired myelin in adult rodents can be formed with less cholesterol than during development. Future studies with isotope labeling to track cholesterol biosynthesis and metabolism during drug treatment would better inform our interpretation of these data. It will also be valuable to extend these findings to other preclinical models of demyelination and human multiple sclerosis tissue samples. 

Although cholesterol esters are not highly abundant in healthy CNS tissue (<1% of total cholesterol) [8], accumulation of cholesterol esters has been observed in postmortem CNS tissues from patients with myelin damage [15], and in cerebrospinal fluid of MS patients with more severe symptoms [16]. Cholesterol esters also accumulate in the CNS in animal models of demyelination [15]. Cholesterol ester levels in the brain increased during demyelination and decreased with remyelination in the *Plp1*-iCKO-*Myrf* model [20]. To analyze how the measured cholesterol pool changed in the model, we compared the cholesterol and cholesterol ester levels in the brain and spinal cord during peak demyelination. At peak demyelination, we observed that brain cholesterol esters comprise 19% of the measured cholesterol pool, as compared to 1% in healthy brains (Figure 5C, Supplementary Table S11). With demyelination in the spinal cord, the total cholesterol esters increased to 66% at peak demyelination. Similar to the healthy brain, the adult healthy mouse spinal cord had 1 nmol/mg cholesterol esters, accounting for 1.4% of the measured cholesterol pool (Figure 5D, Supplementary Table S12). Our data indicate that there is substantial accumulation of cholesterol esters during demyelination, which represents a large potential pool of cholesterol that could be stored for possible recycling and remyelination. However, significant questions remain. The cellular localization of the cholesterol esters is not yet clear, as we previously observed overexpression of both ACAT1 and LCAT [20], which suggests that there may be intracellular accumulation of cholesterol esters in lipid droplets and extracellular accumulation in apolipoprotein particles.

The difference between the brain and spinal cord in the *Plp1*-iCKO-*Myrf* mice in terms of cholesterol esters is especially striking. Our previous data showed that brain cholesterol esters return to normal levels during remyelination, but that the spinal cord showed chronic accumulation of cholesterol esters and limited remyelination [20]. Here, we have determined that more than half of the measured cholesterol pool (66%) in the spinal cord consists of cholesterol esters. We have recently observed that the microglia in the brain and spinal cord have a heterogeneous response to demyelination in the *Plp1*-iCKO-*Myrf* mice [20,24]. Spinal cord microglia have a more disease-associated amoeboid morphology and adopt a pro-inflammatory state. In contrast, brain microglia are more quickly activated and show a greater upregulation of markers associated with phagocytosis and myelin debris clearance. Future mechanistic studies are needed to determine why the spinal cord microglia behave differently or whether chronic accumulation of cholesterol esters in the spinal cord directly slows remyelination. 

## 4. Materials and Methods

### 4.1. Animal Studies

All procedures were approved by the University of Kansas Institutional Animal Care and Use Committee (Animal Welfare Assurance number D16-00220 (A3339-01)). Mice were housed in 12 h/12 h light/dark cycles with ad libitum chow. A genetic mouse model of demyelination with a C57BL6/J background was utilized in this study where *Myrf(fl/fl)* mice were crossed with *Plp1-CreERT* mice to generate the *Plp1-CreERT*; *Myrf (fl/fl)* strain (or *Plp1*-iCKO-*Myrf*) at the University of Kansas [29,30,55,56]. For the demyelination study, all mice at 8 weeks of age received intraperitoneal injections of tamoxifen (Thermofisher, Ward Hill, MA, USA, AAJ6350903) for five days (100 µL of 20 mg/mL daily in corn oil) to induce Myrf ablation and demyelination. Mice with *CreERT* (or *Cre*-positive) lose Myrf in oligodendrocytes and undergo demyelination, and those without *CreERT* (or *Cre*-negative) serve as healthy controls. Genotyping was performed using PCR as previously described [30]. Mice were randomly assigned to five time points and euthanized (carbon dioxide inhalation and cervical dislocation) at 6, 12, 18, 24, and 30 weeks post-tamoxifen injection. Based on prior experience with the model [23,31], power analysis assuming a 2-fold effect size, and a desire to have both sexes represented, we planned to enroll 6–8 mice per genotype at each time point. Final mouse numbers for the experiment were as following: week 6 *Cre*-negative n = 6, week 18 *Cre*-positive n = 8, week 12 *Cre*-negative n = 5, week 12 *Cre*-positive n = 7, week 18 *Cre*-negative n = 6, week 18 *Cre*-positive n = 8, week 24 *Cre*-negative n = 3, week 24 *Cre*-positive n = 7, week 30 *Cre*-negative n = 7, and week 30 *Cre*-positive n = 9. A mix of male and female mice was used in all groups and the exact numbers are provided in Supplementary Tables S3–S6. All groups contained mice from multiple litters. Brain and spinal cord tissues were collected and stored at −80 °C. Additionally, a randomly chosen cohort of *Cre*-negative and *Cre*-positive mice from weeks 12 and 18 post-tamoxifen received Sob-AM2 chow treatment starting 2 weeks post-tamoxifen. Sob-AM2 was compounded into chow (Envigo Teklad 2016 diet) at a dose of 420 µg/kg chow (a nominal daily dose of 84 µg/kg body weight). Final mice numbers for the experiment were the following: week 12 Sob-AM2 *Cre*-negative n = 6, week 12 Sob-AM2 *Cre*-positive n = 10, week 18 Sob-AM2 *Cre*-negative n = 4, week 18 Sob-AM2 *Cre*-positive n = 9.

In the developmental experiment, mice from the *Plp1*-iCKO-*Myrf* strain were used and included a mix of *Cre*-negative and *Cre*-positive mice, but no tamoxifen was administered to these mice. The mice were not genotyped, and all mice expressed *Myrf* normally at all time points (P1, P4, P7, P10, P14, P21, and P42). For each group, n = 3 mice were used, except for the brain at P1 (n = 5) and the spinal cord at P1 (n = 4). A mix of male and female mice was used in all groups. The exact numbers are provided in Supplementary Tables S1 and S2.

Animals were excluded from the study if they died prematurely during the course of the study. Motor ability was monitored during the development of demyelination. At peak demyelination, mice showed moderate hindlimb paresis, which resolved by week 18. Removal from the study would occur if mice exhibited severe hindlimb paresis or were inactive with hunched posture and elevated heart rate; however, no adverse events were observed during this study. Confounders like animal/cage location were not controlled. The protocol was not registered prior to the study. Scientists were not blinded to genotype or time point during the in vivo phase of the study. However, scientists were blinded to genotype during the mass spectrometry experiment and data analysis.

### 4.2. Modified Bligh & Dyer Lipid Extraction Protocol for the Brain Homogenates of Healthy and Demyelinating Mice

Brain tissues were homogenized (Bead Mill Homogenizer, OMNI International, Kennesaw, GA, USA, 19-2241E) at 50 mg/mL or 300 mg/mL (10 ceramic beads (OMNI International, 19-645-3) at a speed of 3.10 m/s for 3 cycles of 20 s, including a 1 s dwell time. Spinal cord tissue was homogenized at 65 mg/mL (3 ceramic beads, 4.0 m/s, 2 cycles of 20 s, 1 s dwell time) in ice-cold micro pure water. The brain and spinal cord tissue homogenates were aliquoted and stored at −80 °C.

Due to the large number, samples were randomized into five batches (n = 15–20 in each batch) to perform modified Bligh & Dyer lipid extraction [57]. Brain homogenates (6 mg tissue total amount) were diluted to 6 mg/mL in 1 mL of ice-cold micro pure water. Each sample was vortexed well, and 10 µL from each sample was saved for protein quantification using BCA assay if needed. The remaining 990 µL homogenate was added into glass tubes (VWR International, Secaucus, NJ, USA, 72690-022) containing CHCl_3_ (with 0.01% butylated hydroxytoluene, BHT):MeOH:water in a 3:2:1 ratio. Then, 5 µL of 2.54 mM d_7_-cholesterol in CHCl_3_ (Cayman Chemicals, Ann Arbor, MI, USA, 25265) was spiked into each tube. The glass tubes were shaken and vortexed to allow mixing. Then, 2.5 mL of CHCl_3_ was added to each glass tube, followed by the addition of 1.25 mL of water. Again, the glass tubes were shaken and vortexed ten times and five times, respectively. The samples were centrifuged (ThermoScientific Sorvall ST40R, Osterode am Harz, Germany, 42508141) at 400× *g* for 10 min at 25 °C. Then, the lower CHCl_3_ layer was removed into a small glass tube (VWR International, 72690-024), and the extractions were repeated with another 1.25 mL of CHCl_3_ for two more times. All three extracted CHCl_3_ layers were combined and washed with 300 µL of 1 M KCl and 300 μL of water, respectively (centrifuged at 400× *g* for 5 min at 25 °C). The CHCl_3_ layer was vacuum dried (ThermoScientific Savant, Asheville, NC, USA, SPD130DLX). Then, 0.5 mL of CHCl_3_ was used to resuspend the lipids and transferred to PTFE-capped glass vials for storage. Finally, the samples were vacuum dried again, weighed, and stored at −80 °C in the freezer. The extracted total lipid amount was resuspended in 100 µL and 10 µL (10%) was used for the derivatization reaction.

### 4.3. Modified Bligh & Dyer Lipid Extraction Protocol for Spinal Cord Homogenates of the Healthy and Demyelinating Mice

Spinal cord samples were also randomized (four batches) prior to the lipid extraction. The modified Bligh and Dyer protocol as described above was followed for the spinal cord homogenates with the following exceptions: spinal cord homogenates (3.25 mg tissue total amount) were diluted to 3.25 mg/mL in 1 mL of ice-cold micro pure water, and 100 µL was saved for the BCA assay. The remaining 900 µL was used for the extraction, and 10 µL of d_7_-cholesterol (1 mM standard) was spiked into each tube. The extracted total lipid amount was resuspended in 100 µL, and 20 µL (20%) was used for the derivatization reaction.

### 4.4. Lipid Extraction Protocol for Postnatal Mouse Brain and Spinal Cord Homogenates

The same lipid extraction protocol was followed for the postnatal mouse brain (5 mg of brain tissue in 1 mL ice-cold micro pure water) and spinal cord (2.5 mg tissue in 1 mL ice-cold micro pure water). The spiked amounts of d_7_-cholesterol (1 mM standard) were 15 µL and 20 µL for the brain and spinal cord, respectively. The extracted total lipid amount was resuspended in 100 µL, and 20 µL (20%) was used for the derivatization reaction.

### 4.5. Sample Derivatization for the GC-MS

Extracted healthy and demyelinating total lipid extracts (both brain and spinal cord) were resuspended in 100 µL CHCl_3_ (0.01% BHT) and sonicated (Branson M2800, Danbury, CT, USA) for 15 min. Then, respective volumes of each sample (as described above) were transferred into reaction vials. Gas-tight syringes (Hamilton, Reno, NV, USA) were used for transferring all organic solvents throughout the protocol. The reaction vial wall was washed with 300 µL of CHCl_3,_ and then the CHCl_3_ was vacuum evaporated. Next, 60 µL of N, O-bis(trimethylsilyl)trifluoroacetamide with trimethylchlorosilane (BSTFA + TMCS, 99:1, *v*/*v*) derivatizing reagent (LiChropur, Merck KGaA, Allegheny County, PA 15238, USA) was added and incubated at 50 °C for 1 h. Samples were cooled to room temperature, and 140 µL of n-hexane was added. Then the reaction mixtures were vortexed and transferred into glass vials (ThermoScientific, C4013-2) with glass inserts (Supelco, Merck KGaA, Mercer County, WV 24701, USA) and 1 µL of each mixture was injected into the GC-MS. (Note: The trimethylsilyl derivatized cholesterol can degrade after 48 h at room temperature. Therefore, all experiments for each batch were performed over two days following the same timeline.)

### 4.6. Preparation of Calibration Curves

Standard solutions of d_0_-cholesterol (0.50 mM) and d_7_-cholesterol (2.54 mM) were prepared using the solvent CHCl_3_ (0.01% BHT). BSTFA + TMCS, 99:1 *v*/*v*, reagent was used to derivatize standards following the protocol described above. Derivatized standards were dissolved in n-hexane before GC-MS injections. Separate calibration curves were obtained for each batch of samples. For the brain calibration curve, cholesterol standards were in the 4.0–80.0 µM range with a d_7_-cholesterol spike of 12.70 µM, while spinal cord calibration curve standards were in the range of 4.0 µM–0.16 mM range with a d_7_-cholesterol spike of 8.0 µM (Supplementary Table S13). For cholesterol quantification, analyte recovery and matrix effects were not assessed.

### 4.7. Quality Controls

Quality controls (QCs) were prepared for the brain and spinal cord by mixing equal volumes of representative samples selected from each time point, including both Cre-positive and Cre-negative mouse groups. The mixtures were vortexed well and diluted to a final concentration of 50 mg/mL for brain samples and 65 mg/mL for spinal cord samples. The quality control mixtures were aliquoted, and three aliquots per batch were extracted, derivatized, and analyzed by GC-MS alongside the other samples in the batch. Only a single QC concentration was measured, and two to three quality controls were incorporated for each batch (Supplementary Table S13). Acceptance criteria for the analytical batches were not defined prior to the experiments. However, the CV values for both brain and spinal cord QCs were less than 15%, which fell within the acceptance criteria recommended for bioanalytical assays [32].

### 4.8. Gas Chromatography-Mass Spectrometry (GC-MS) Conditions and Data Analysis

Gas chromatography–mass spectrometry was performed using a capillary SH-I-5MS Column (0.25 mm, 0.25 µm film, 30.0 m) on a Shimadzu QP2010 SE (Columbia, MD, USA). Helium was used as a carrier gas at a flo wrate of 1 mL/min. The initial column oven temperature was held at 150 °C and the injection temperature was set to 290 °C. The initial temperature of 150 °C was ramped to 305 °C at 30 °C/min and then ramped at 1 °C/min to 315 °C with a hold of 1 min at 315 °C. The total run time was 17.17 min. Electron impact ionization was performed at 70 eV with ion source temperature of 230 °C and an interface temperature of 280 °C. The sample was injected at a volume of 1 µL in splitless mode. Blank samples were included between runs to ensure no sample carryover. GC-MS detection was set to single ion monitoring mode. Reproducible fragments (*m*/*z* 329 and *m*/*z* 336) were selected for the quantification of d_0_-cholesterol and d_7_-cholesterol, respectively (Figure 1A) [25]. Peak areas of extracted ion chromatograms were automatically integrated using post-run GCMSsolutions Software (Shimadzu Cooperation, v 4.53).

### 4.9. Lipid Extraction and Liquid Chromatography–Mass Spectrometry Protocol for Cholesterol Ester Levels in Mouse Brain and Spinal Cord

To measure cholesterol esters in developmental tissues, 15 mg of developmental brain tissue and 5 mg of developmental spinal cord tissue were diluted in 1 mL of ice-cold micro pure water and the modified Bligh and Dyer lipid extraction was followed. The developmental brain and spinal cord tissues were spiked with 15 μL and 20 μL of 200 μM d_9_-cholesterol palmitate (Cayman, Ann Arbor, MI, USA, 28123), respectively. Brain total lipid extract was resuspended in 300 μL of 2MeOH:CHCl_3_. The resuspended extracted total lipid amount was diluted 2.5 times (100 μL was taken out, evaporated and resuspended in 250 μL) for LC-MS injection. Spinal cord lipid extract was dissolved 100 μL of 2MeOH:CHCl_3_ and diluted 1.5 times (100 μL was taken out, evaporated and resuspended in 150 μL) for the injection. A calibration curve was prepared using a range of 2.00–26.25 μM of d_0_-cholesterol palmitate (Cayman, 26473), each containing a 7.5 μM spike of d_9_-cholesterol palmitate. For cholesterol ester quantification, analyte recovery and matrix effects were not assessed. To measure cholesterol esters in adult CNS tissues, 15 mg of adult healthy and demyelinated brain tissue, 6.5 mg of healthy spinal cord and 3.9 mg of demyelinated spinal cord were used in diluted 1 mL ice-cold micro pure water. Modified Bligh and Dyer lipid extraction was performed with 200 μM d_9_-cholesterol palmitate spike for healthy and demyelinated brain tissue (40 μL), healthy spinal cord tissue (10 μL) and demyelinated spinal cord tissue (40 μL) respectively. Each total lipid extract was resuspended in 300 μL of 2:1 MeOH:CHCl_3_ and diluted 10 times (20 μL was taken out, evaporated and resuspended in 200 μL) for LC-MS injection. Calibration curves were prepared for adult brain and spinal cord batches in the range of 2.00–26.25 μM of d_0_-cholesterol palmitate (Cayman, 26473), each containing a 7.5 μM spike of d_9_-cholesterol palmitate. High-resolution LC-MS experiments were performed using an Orbitrap Fusion (Milford, MA, USA). Mobile phases consisted of solvent A: ACN/H_2_O (40:60, *v*/*v*) and solvent B: H_2_O/ACN/IPA (1:9:90, *v*/*v*) both containing 10 mM ammonium formate. Five microliters of each sample were injected onto a T3 HSS C18 (3.5 μm, 1 mm, 150 mm) at a flow rate of 50 μL/min. The lipids were separated using a 15 min gradient starting at 30% B, then a 1.5 min increase to 65% B, then ramped to 99% B in 1.5 min, held for 2.0 min, and then returned to initial conditions (30% B) for 6 min. The column was re-equilibrated at initial conditions for 4 min prior to each new injection. A short wash and a blank run were performed between every sample to ensure no sample carryover. Cholesterol esters were eluted within 7.90–10.15 min time frame in a total run time of 15 min. Mass spectrometric analysis was performed in the positive ion mode using the following parameters: a spray voltage of 3.0 kV, sheath gas: 10 units, auxiliary gas: 6 units, ion transfer tube maintained at 250 °C, and the vaporizer set to 60 °C. Full MS scans in the Orbitrap were performed in 1.5 s cycles in the mass range of *m*/*z* 300–1200 at a resolution of 120,000 at *m*/*z* 200 with an automatic gain control target value set to 4 × 10^5^ and with a maximum injection time of 50 ms. Peak intensities (S/N ≥ 10 with mass error less than ±2 ppm) of ammonium adducts of twenty-one cholesterol ester types (Supplementary Tables S7–S12) were manually integrated using Xcalibur software (Version 2.2.0.48). The enviPAT isotope pattern calculator was used to determine the *m*/*z* values for the monoisotopic cholesterol esters and the respective ammonium adducts. This method was not able to distinguish different cholesterol ester isomers. 

### 4.10. Statistics and Data Analysis

Internal standard-based calibration curves were used to calculate the cholesterol level in both brain and spinal cord tissues. Cholesterol levels were normalized to tissue weight for both brain and spinal cord. Data were plotted as mean ± SEM. 

Total cholesterol levels in the P1–P42 developmental time points were compared using ordinary one-way ANOVA. The Brown–Forsythe test confirmed that the standard deviations were not significantly different. The Shapiro–Willk test confirmed that all groups showed normality of distribution, except for P7 in the spinal cord. No outliers were detected or excluded from the analysis. Dunnett’s multiple comparisons test was performed comparing P1 to all other time points. *p* value is adjusted for multiple comparisons with a 95% confidence interval, where ns ≥ 0.05, * *p* < 0.05; ** *p* < 0.01; *** *p* < 0.001; **** *p* < 0.0001. Exact statistical parameters for all tests are presented in Supplementary Tables S14 and S15.

Total cholesterol esters for each developmental sample were calculated by summing the individual species. Total cholesterol esters were plotted as mean ± SEM and were compared using ordinary one-way ANOVA. The Brown–Forsythe test confirmed that the standard deviations were not significantly different. The Shapiro–Willk test confirmed that all groups showed normality of distribution except for P14 in the spinal cord. No outliers were detected or excluded from the analysis. Dunnett’s multiple comparisons test was performed comparing P1 to all other time points. *p* value is adjusted for multiple comparisons with a 95% confidence interval, where ns ≥ 0.05, * *p* < 0.05; ** *p* < 0.01; *** *p* < 0.001; **** *p* < 0.0001. Exact statistical parameters for all tests are presented in Supplementary Tables S16 and S17.

Total free cholesterol levels in the demyelinated mouse brain and spinal cord were compared using a two-way ANOVA. Significant group effects were observed for genotype and time in both the brain and spinal cord. An interaction effect for genotype/time was only observed in the brain. Tukey’s multiple comparisons test was used to compare genotypes within each time point and all time points within each genotype, where ns ≥ 0.05, * *p* < 0.05; ** *p* < 0.01; *** *p* < 0.001; **** *p* < 0.0001. Exact statistical parameters for all tests are in Supplementary Tables S18 and S19.

The effect of the Sob-AM2 drug on cholesterol levels in the brain and spinal cord was evaluated by a three-way ANOVA. Significant group effects were observed for genotype and drug treatment in the brain and only genotype in the spinal cord. No group interaction effects were observed. Šídák’s multiple comparisons test was used to compare groups that only varied by one factor, where ns ≥ 0.05, * *p* < 0.05; ** *p* < 0.01; *** *p* < 0.001; **** *p* < 0.0001. Exact statistical parameters for all tests are in Supplementary Tables S20 and S21.

A subset of the demyelinated samples were analyzed for cholesterol esters with n = 3 for brain at week 12 and spinal cord at week 18, which represent the peak cholesterol ester accumulation as previously described [20]. An unpaired *t*-test was used to compare genotypes, where ns ≥ 0.05, * *p* < 0.05; ** *p* < 0.01; *** *p* < 0.001; **** *p* < 0.0001. Exact statistical parameters are in Supplementary Tables S22 and S23. 

Total amounts of cholesterol ester (CE) and individual CE types are included in heat maps as average CE nmol amount per mg tissue and all data are included in Supplementary Tables S7–S12.

Statistical analysis was performed using GraphPad Prism 11 and figures were prepared using BioRender, GraphPad Prism 11, and Adobe Illustrator v30.4. Raw mass spectrometry files will be available in the Metabolomics Workbench repository (accession number ST005074). 

## 5. Conclusions

In this study, we developed a robust platform of analytical methods using internal standards to quantify CNS tissue levels of cholesterol and cholesterol esters, using GC-MS-SIM and LC-MS, respectively. We used these methods to measure how brain and spinal cord cholesterol levels fluctuate over 12 cross-sectional time points during postnatal myelination, demyelination, and remyelination in an inducible genetic mouse model. Our measurements highlight key differences between the brain and spinal cord, including the higher concentration of cholesterol in the spinal cord, the continuous increase in brain cholesterol up to 10 months of age, and cholesterol ester dynamics during demyelination. Future studies will focus on revealing the mechanisms behind the chronic loss in cholesterol and elevation of cholesterol esters during demyelination. Identifying targets to preserve CNS cholesterol or restore cholesterol levels could be a promising therapeutic strategy for neurodegenerative diseases and age-related myelin degeneration.

## Data Availability

All analyzed mass spectrometry data is available with the manuscript or the Supplementary Information. Raw mass spectrometry files will be available in the Metabolomics Workbench repository (accession number ST005074).

## Supplementary Materials

The following supporting information can be downloaded at: https://www.mdpi.com/article/10.3390/molecules31173033/s1, Supplementary Table S1: Developmental free cholesterol level in the mouse brain P1–P42; Supplementary Table S2: Developmental free cholesterol level in the mouse spinal cord P1–P42; Supplementary Table S3: Free cholesterol level in the adult healthy and demyelinated mouse brains; Supplementary Table S4: Free cholesterol level in the adult mouse brains with Sob-AM2 drug treatment; Supplementary Table S5: Free cholesterol level in the adult healthy and demyelinated mouse spinal cords; Supplementary Table S6: Free cholesterol level in the adult mouse spinal cords with Sob-AM2 drug treatment; Supplementary Table S7: Cholesterol ester amounts in the developmental brain P1–P42; Supplementary Table S8: Cholesterol ester percentage over measured cholesterol pool in the developmental brain P1–P42; Supplementary Table S9: Cholesterol ester amounts in the developmental spinal cord P1–P42; Supplementary Table S10: Cholesterol ester percentage over measured cholesterol pool in the developmental spinal cord P1–P42; Supplementary Table S11: Cholesterol ester amounts in healthy and demyelinated brain at peak demyelination; Supplementary Table S12: Cholesterol ester amounts in healthy and demyelinated spinal cord at peak demyelination; Supplementary Table S13: Respective calibration curves utilized for mass spectrometry runs; Supplementary Table S14: Statistical parameters for developmental free cholesterol level in the mouse brain P1–P42; Supplementary Table S15: Statistical parameters for developmental free cholesterol level in the mouse spinal cord P1–P42; Supplementary Table S16: Statistical parameters for total cholesterol esters in the mouse brain P1–P42; Supplementary Table S17: Statistical parameters for total cholesterol esters in the mouse spinal cord P1–P42; Supplementary Table S18: Statistical parameters for free cholesterol level in the demyelinated mouse brain; Supplementary Table S19: Statistical parameters for free cholesterol level in the demyelinated mouse spinal cord; Supplementary Table S20: Statistical parameters for free cholesterol level in the demyelinated mouse brain with Sob-AM2 treatment; Supplementary Table S21: Statistical parameters for free cholesterol level in the demyelinated mouse spinal cord with Sob-AM2 treatment; Supplementary Table S22: Statistical parameters for cholesterol esters in the demyelinated mouse brain; Supplementary Table S23: Statistical parameters for total cholesterol esters in the demyelinated mouse spinal cord.

## Abbreviations

The following abbreviations are used in this manuscript:

CNS	Central nervous system
GC-MS	Gas chromatography–mass spectrometry
SIM	Single ion monitoring
LC-MS	Liquid chromatography–mass spectrometry
P	Postnatal day
PTI	Post-tamoxifen injection
CE	Cholesterol esters
MS	Multiple sclerosis
Sob-AM2	Sobetirome-amide-2
QC	Quality control
